# Genetic Deficiency of the Macrophage Csf2ra Receptor Modulates Inflammatory Responses Following Cardiac Ischaemic Injury in Mice

**DOI:** 10.3390/cells15090764

**Published:** 2026-04-24

**Authors:** Georgios Kremastiotis, Yong Li, Andrew Bond, Daire Shanahan, Karina Di Gregoli, Alastair W. Poole, Sarah J. George, Jason L. Johnson

**Affiliations:** Laboratory of Cardiovascular Pathology, Bristol Medical School, Faculty of Health & Life Sciences, University of Bristol, Bristol BS2 8HW, UK; giorgoskrt@gmail.com (G.K.); yong.li@bristol.ac.uk (Y.L.); andrew.bond@bristol.ac.uk (A.B.); daire.shanahan@bristol.ac.uk (D.S.); karina.digregoli@gmail.com (K.D.G.); a.poole@bristol.ac.uk (A.W.P.); s.j.george@bristol.ac.uk (S.J.G.)

**Keywords:** cardio-immunology, Csf2ra, GM-CSF, genetic deficiency, macrophages, inflammation

## Abstract

Myocardial infarction (MI) triggers a robust inflammatory response that is essential for tissue repair but, when excessive or prolonged, drives pathological cardiac remodelling and heart failure. Colony-stimulating factor 2 (CSF2) signalling has been implicated in driving pro-inflammatory macrophage activation post-MI. Here, we investigated the role of macrophage-specific CSF2 receptor alpha (CSF2RA) signalling in post-MI remodelling using a tamoxifen-inducible genetic mouse model and permanent coronary artery ligation. Macrophage-specific Csf2ra deficiency significantly improved left ventricular systolic function post-MI without altering cardiac fibrosis burden. Functional improvement was associated with enhanced collagen scar maturation, characterised by an increased proportion of mature collagen fibres, and with accumulation of anti-inflammatory, pro-reparative macrophages within the infarct. These macrophage changes were accompanied by increased fibroblast density, consistent with altered macrophage–fibroblast crosstalk. Collectively, these findings identify macrophage-intrinsic CSF2RA signalling as a critical regulator of inflammatory resolution and scar maturation after MI and provide mechanistic support for the rationale of selective CSF2RA inhibition as a therapeutic strategy to limit adverse cardiac remodelling and improve post-infarction recovery.

## 1. Introduction

Myocardial infarction (MI) has an overall prevalence of 4.5% for males and 2.1% for females and accounts for over 605,000 incidents each year in the United States alone [1]. Approximately one out of three patients who survive an MI subsequently develop heart failure, profoundly impairing their quality of life and long-term survival [2,3,4]. Recovery following MI is not determined solely by infarct size but is critically influenced by the structural quality and organisation of the reparative scar. Human contrast-enhanced cardiovascular magnetic resonance studies demonstrate that heterogeneity within the infarct and peri-infarct regions reflects differences in scar composition and organisation that have important functional consequences [5,6]. Although fibrotic scar formation is essential to preserve ventricular integrity after infarction, variability in scar architecture is associated with altered mechanical and electrical properties of the myocardium, contributing to impaired ventricular compliance, inefficient contraction, and adverse remodelling [5,7]. These findings underscore that the maturation and structural organisation of post-MI scar tissue play a central role in determining long-term functional recovery and clinical outcome, independent of total infarct burden [6]. Impaired scar formation, often driven by non-resolving inflammation, disrupts scar maturation and organisation and contributes to ventricular dilatation and progressive heart failure [8,9]. Accordingly, defining the molecular drivers that govern inflammatory responses and scar formation following acute ischaemic injury represents a critical step in identifying translational targets that promote post-MI recovery and limit subsequent heart failure risk.

Monocyte-derived macrophages are central coordinators of the inflammatory response and scar remodelling post-MI [10,11]. Monocytes are mobilised from the circulation and splenic reservoir in a C-C chemokine receptor type 2- (CCR2-)dependent manner and accumulate within the infarct, where they differentiate into macrophages under the influence of an intricate inflammatory milieu [12,13]. Beyond debris clearance, macrophages critically shape fibroblast behaviour and may directly contribute to extracellular matrix (ECM) synthesis within the healing myocardium [10,14,15]. Dysregulation of macrophage activation or persisting inflammation has been causally linked to defective scar maturation and adverse ventricular remodelling [16].

Colony-stimulating factors (CSFs) are key regulators of macrophage differentiation and functional programming within tissues [17]. Granulocyte macrophage colony-stimulating factor (GM-CSF, CSF2) is secreted by the expanding cardiac fibroblast population and is upregulated in the ischaemic myocardium [18,19]. CSF2 has been associated with sustained macrophage activation and inflammatory signalling [20]. CSF2 signals through a receptor complex comprising a ligand-specific alpha chain (CSF2RA) and a shared beta chain (CSF2RB), which is also required for interleukin IL-3 and IL-5 signalling [21]. Previous studies targeting the CSF2/CSF2R axis in post-MI have relied on mouse models with genetic Csf2 or Csf2rb deficiency, demonstrating a clear beneficial effect on inflammation resolution and cardiac remodelling [18]. However, these approaches are limited in their ability to delineate between macrophage-specific CSF2 effects and effects driven by other CSF2RB-dependent cytokines such as IL-3 and IL-5 (Csf2rb^−/−^) or neutrophils (Csf2^−/−^). We recently employed a selective pharmacological inhibitor of CSF2RA to explore a direct role for CSF2-polarised macrophages in pathological cardiac remodelling and showed that targeted interference with CSF2RA improves post-MI cardiac remodelling and modulates macrophage and fibroblast intercellular communication during the early inflammatory and proliferative stages [22].

In the present study, we selectively ablated Csf2ra in macrophages using a tamoxifen-inducible genetic model, thereby isolating the macrophage-intrinsic contribution of Csf2ra signalling to post-MI repair and providing mechanistic support to strengthen our prior scientific evidence [22] supporting the therapeutic strategy of utilising CSF2RA inhibition. We show that macrophage-specific loss of Csf2ra is associated with an early improvement in left ventricular function, partly by promoting the accumulation of macrophages with reparative-associated marker enrichment and modifying collagen scar maturation during late-stage remodelling. Together, these findings reveal macrophage-intrinsic CSF2RA signalling as a key regulator of inflammatory resolution, scar maturation, and functional recovery post-MI.

## 2. Materials and Methods

### 2.1. Generation of Mice with Inducible Monocyte/Macrophage Cell–Specific Loss of Csf2ra

We bred Csf1r-Cre/Esr1 mice (The Jackson Laboratory, Bar Harbor, ME, USA; stock no. 019098) with Csf2ra-floxed mice (PolyGene AG, Rümlang, Switzerland) to generate tamoxifen-inducible monocyte/macrophage-specific deletion of Csf2ra (Csf2ra mac-KO) and the corresponding Csf2ra^fl/fl^ and Csf1r-Cre^−ve^ littermate controls (Csf2ra mac-WT). Male and female mice were injected intraperitoneally daily for 5 days with tamoxifen (20 mg/mL) to induce monocyte/macrophage-specific deletion of Csf2ra 7 days before surgery.

### 2.2. Cardiac Dysfunction and Fibrosis Model in Mice

Mice were subjected to a refined model of ischaemic injury via permanent ligation of a distal portion of the LAD, resulting in smaller infarcts, markedly improved survival, and a reduction in the number of mice used in research, in compliance with the Animals in Science Regulation Unit (ASRU) and NC3Rs guidance, as previously described [22]. Additionally, we have previously validated that the refined model of LAD ligation resulted in deterioration in cardiac function, which was associated with increased macrophage accumulation, myofibroblast activation, and cardiac fibrosis, indicative of a wound healing response to ischaemic injury, as opposed to the surgical sham control group [22]. In line with the ARRIVE guidelines around reducing the number of animals used in research, we did not repeat the surgical sham control group for this study. Briefly, mice were anaesthetised with isoflurane (2.5% in oxygen, 1 L/min), intubated, and mechanically ventilated (MiniVent Model 845, 73-0044, Hugo Satchs Electronik, March-Hugstetten, Gernany). Following incision of the thorax and pericardium, the LAD was ligated with an 8-0 Ethilon suture, and the chest and skin were closed before recovery. Mice received analgesia (buprenorphine, Temgesic, Eumedica, Manage, Belgium; 0.1 mg/kg) and were closely monitored for 24 h after surgery. Cardiac function was assessed by echocardiography (Vevo 3100) and analysed using Vevo LAB 5.7.1 (Fujifilm VisualSonics, Amsterdam, The Netherlands). To note, the echocardiography WT control data in Table 2 were also utilised in our previous study as a control group [22], as the studies were run concurrently. At 28 days post-surgery, mice were culled with sodium pentobarbitone (500 mg/kg, i.p.) and perfused via the abdominal aorta with PBS (100 mmHg), followed by 10% formalin. Hearts were processed for histology and 3-μm sections were cut for downstream analyses. The researchers remained blinded to the genotype.

### 2.3. Histochemistry

Myocardial fibrosis was assessed using a Masson’s trichrome staining kit (HT15, Sigma-Aldrich, Gillingham, UK) and fibrillar collagen fibre content was assessed using a Picrosirius Red stain kit (ab245887, Abcam, Cambridge, UK), according to the manufacturer’s instructions. H&E staining was performed using a Shandon Varistain 24-4 Automatic Slide Stainer (74200103, Thermo Fisher Scientific, Birmingham, UK). Specific marker expression and localisation were assessed by immunofluorescence. Briefly, slides were dewaxed, rehydrated, treated to block endogenous peroxidase activity, and subjected to heat-mediated antigen retrieval in citrate buffer (pH 6.0). Sections were blocked in serum-free blocking reagent (GTX30963, GeneTex, Irvine, CA, USA), incubated with primary antibodies (Table 1) with matched IgG isotype controls as negative controls, and detected using fluorophore-conjugated secondary antibodies and DAPI-containing mounting medium; mouse-on-mouse staining was performed using a commercial kit (FMK-2201, 2BScientifc Ltd., Oxfordshire, UK). Cellular composition in the infarct area was quantified and averaged across three sections per mouse, with results normalised to total nuclei or to specific cell populations as indicated. The results of proliferation (PCNA^+^) and expression of cell markers (CD206^+^, iNOS^+^, CXCL10^+^, CTSZ^+^, α-SMA^+^) are expressed as the number of double-positive cells normalised to the total number of macrophages (CD68^+^) or fibroblasts (vimentin^+^).

## 3. Results

### 3.1. Macrophage-Specific Csf2ra Deficiency Improves Cardiac Function and Alters Scar Collagen Content Post-Myocardial Infarction

**Figure 1:**
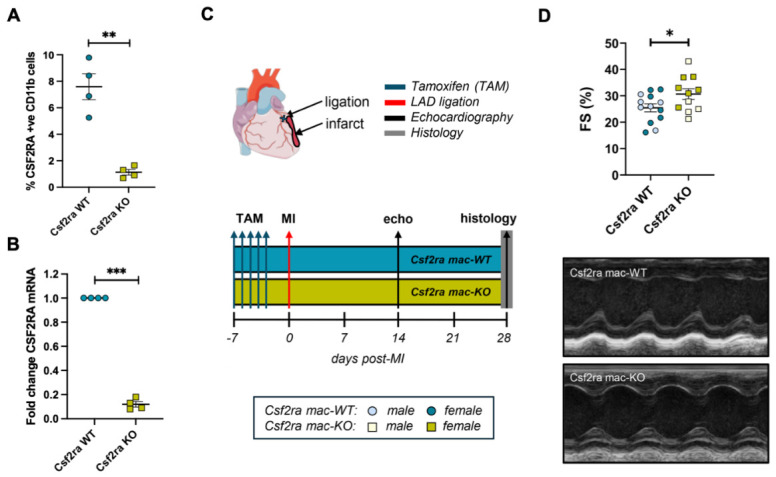
Macrophage-specific Csf2ra deficiency improves cardiac function post-myocardial infarction. (**A**) Quantification of flow cytometry analysis for CSF2RA expression upon CD11b-gated PBMCs (*n* = 4/group). (**B**) QPCR analysis for CSF2RA mRNA expression in adherent monocytes (*n* = 4/group). (**C**) Experimental schematic of Csf2ra mac-KO and littermate WT mice subjected to permanent left anterior descending (LAD) coronary artery ligation. (**D**) Quantification and representative M-mode images for assessment of fractional shortening (FS%) at 14 days post-MI (WT *n* = 13, KO *n* = 11). Csf2ra mac-WT male mice are presented with pale blue circles and female mice with blue circles; Csf2ra mac-KO male mice are presented with pale mustard squares and female mice with mustard squares. Statistical significance is reported as * *p* < 0.05, ** *p* < 0.01, or *** *p* < 0.001, using an unpaired Student’s *t*-test.

**Figure 2:**
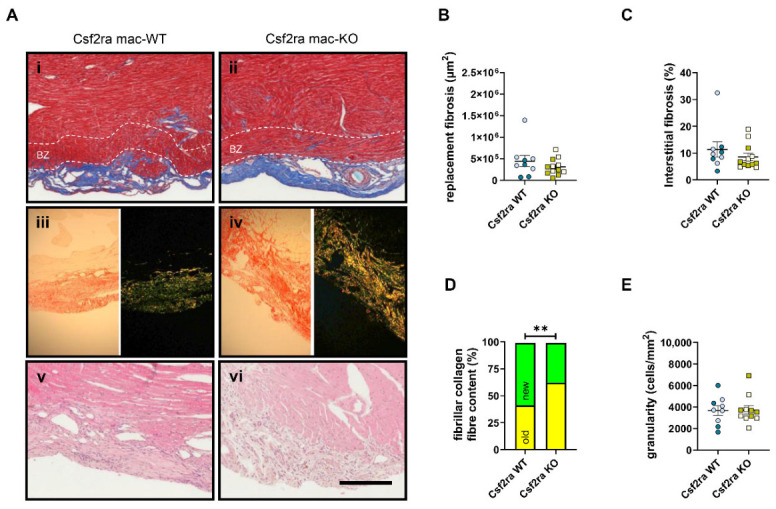
Macrophage-specific Csf2ra deficiency alters scar collagen content post-myocardial infarction. Representative images (**A**(**i**,**ii**)) of cardiac fibrosis from Masson’s trichrome-stained hearts and quantification of (**B**) replacement (μm^2^) and (**C**) interstitial fibrosis (%) in Csf2ra mac-WT (*n* = 9) and -KO mice (*n* = 12). Representative images (**A**(**iii**,**iv**)) of Picrosirius red-stained hearts under brightfield or linearly polarised light and (**D**) quantification of fibrillar collagen fibre content in Csf2ra mac-WT (*n* = 9) and -KO mice (*n* = 12). Representative images (**A**(**v**,**vi**)) of H&E-stained hearts and (**E**) quantification of infarct granularity (cell/mm^2^) in Csf2ra mac-WT (*n* = 9) and -KO mice (*n* = 11). Statistical significance is reported as ** *p* < 0.01 using a Fisher’s exact test in panel (**D**). White dashed lines in images (**A**(**i**,**ii**)) represent the border zone (BZ), defined as a 100-μm region around the replacement fibrosis. The scale bar represents 200 μm and is applicable to all panels. Csf2ra mac-WT male mice are presented with pale blue circles and female mice with blue circles; Csf2ra mac-KO male mice are presented with pale mustard squares and female mice with mustard squares.

To explore a direct role for the Csf2 selective receptor, Csf2ra, in macrophage polarisation in cardiac repair, we utilised male and female mice with tamoxifen-inducible macrophage-specific deficiency of Csf2ra (hereafter referred to as Csf2ra mac-KO). Csf2ra knockdown was confirmed by flow cytometry and QPCR analysis for Csf2ra expression in PBMCs and adherent monocytes, respectively (Figure 1A,B). Csf2ra mac-KO mice and littermate Csf2ra mac-WT controls underwent permanent left anterior descending (LAD) coronary artery ligation, and the hearts were subjected to echocardiography after 14 and 28 days and analysed histologically at 28 days post-MI (Figure 1A). Echocardiography at 14 days post-MI highlighted that macrophage-specific Csf2ra deficiency improved left ventricular function and structure post-MI. Importantly, assessment of fractional shortening (FS) and ejection fraction (EF) from M-mode images revealed a significant improvement in cardiac function in Csf2ra mac-KO mice compared to their Csf2ra mac-WT counterparts (Figure 1B, Table 2). The functional benefit post-MI was accompanied by improved structural characteristics of the left ventricle, highlighted by a significant increase in the left ventricular posterior wall (LVPW) thickness at diastole and systole, in comparison to Csf2ra mac-WT mice, which presented with a thinner wall (Table 2). Echocardiography at 28 days post-MI showed no significant changes between Csf2ra mac-KO mice and littermate, Csf2ra mac-WT controls (Table 3). The hearts of Csf2ra mac-KO and -WT mice were analysed histologically at 28 days post-MI. Csf2ra mac-KO did not affect cardiac fibrosis, as assessed through the area of replacement fibrosis at the infarct (Figure 2A(i,ii),B) or percentage of interstitial fibrosis in the border zone, defined as a 100-μm region around the replacement fibrosis (Figure 2C). However, within hearts from Csf2ra mac-KO, linearly polarised light analysis of Picrosirius red-stained sections demonstrated a significantly greater accumulation of old (yellow) collagen fibres compared to new (green) fibres (Figure 2A(iii,iv),D), suggesting the greater presence of thicker and larger collagen fibrils and indicative of mature collagen as opposed to newly synthesised collagen [23]. Subsequently, we explored the potential effects on granulation tissue and observed no significant changes in infarct granularity following Csf2ra deficiency (Figure 2A(v,vi),E).

### 3.2. Macrophage-Specific Csf2ra Deficiency Alters Infarct Cellular Composition, Increasing Pro-Reparative Macrophage Accrual

**Figure 3:**
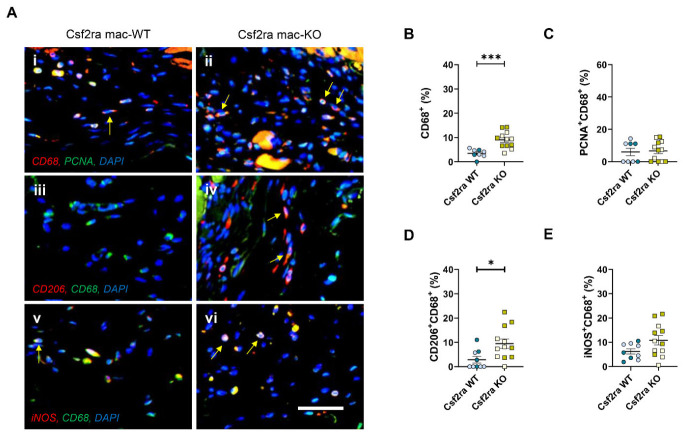
Macrophage-specific Csf2ra deficiency increases pro-reparative macrophage accrual post-myocardial infarction. Representative images of (**A**(**i**,**ii**)) PCNA^+^CD68^+^, (**A**(**iii**,**iv**)) CD206^+^CD68^+^, and (**A**(**v**,**vi**)) iNOS^+^CD68^+^ cells in Csf2ra mac-WT (*n* = 8–9) and -KO mice (*n* = 12). Cells positive for both markers are observed as yellow/white with examples indicated by arrows, with DAPI used as a nuclear marker. Quantification of macrophage (**B**) density, (**C**) proliferation, (**D**) CD206 or (**E**) iNOS protein expression. Statistical significance is reported as * *p* < 0.05 or *** *p* < 0.001, using an unpaired Student’s *t*-test. The scale bar represents 500 μm and is applicable to all panels. Csf2ra mac-WT male mice are presented with pale blue circles and female mice with blue circles; Csf2ra mac-KO male mice are presented with pale mustard squares and female mice with mustard squares.

**Figure 4:**
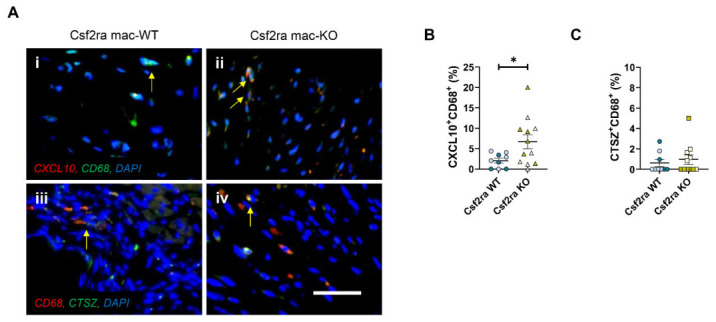
Macrophage-specific Csf2ra deficiency promotes CXCL10-positive macrophages without affecting CTSZ-positive macrophages post-myocardial infarction. Representative images of (**A**(**i**,**ii**)) CD68^+^CXCL10^+^ and (**A**(**iii**,**iv**)) CD68^+^CTSZ^+^ cells in Csf2ra mac-WT (*n* = 9) and -KO mice (*n* = 11–12). Cells positive for both markers are observed as yellow/white with examples indicated by arrows, with DAPI used as a nuclear marker. Quantification of (**B**) CXCL10- and (**C**) CTSZ-positive macrophages. Statistical significance is reported as * *p* < 0.05, using an unpaired Student’s *t*-test. The scale bar represents 500 μm and is applicable to all panels. Csf2ra mac-WT male mice are presented with pale blue circles and female mice with blue circles; Csf2ra mac-KO male mice are presented with pale mustard squares and female mice with mustard squares.

Although the area of cardiac fibrosis (infarct size) and infarct granularity (cell density) were not directly affected, Csf2ra mac-KO affected the cellular composition of infarcts. Firstly, we employed a macrophage analysis by immunohistochemistry to investigate the effects of Csf2ra deficiency on the phenotype of infarct macrophages. Csf2ra mac-KO infarcts contained significantly more macrophages (Figure 3A(i,ii),B) in comparison to Csf2ra mac-WT mice, without changes in macrophage proliferation frequency (Figure 3C), suggesting heightened monocyte/macrophage accumulation. Secondly, in Csf2ra mac-KO mice, macrophage accumulation was associated with enrichment of anti-inflammatory-associated markers, as evidenced by a significant increase in the presence of CD206-positive macrophages within the infarcts (Figure 3A(iii,iv),D), accompanied by non-significant changes in the expression of pro-inflammatory marker iNOS (Figure 3A(v,vi),E). We have previously described the involvement of a cathepsin Z (CTSZ)/CXCL10-mediated mechanism in the effects of CSF2 signalling at days 3, 7, and 14 post-MI [22]. Accordingly, we assessed the protein abundance of CTSZ and CXCL10 within infarct macrophages following macrophage-specific Csf2ra deficiency. In line with the effects of CSF2RA inhibition [22], Csf2ra genetic ablation resulted in a significant increase in CXCL10-positive macrophages within the infarcts of Csf2ra mac-KO mice in comparison to littermate WT controls (Figure 4A(i,ii),B). No significant changes were observed in CTSZ protein abundance (Figure 4A(iii,iv),C). These findings suggest that diminished CSF2 signalling through CSF2RA dictates monocyte/macrophage polarisation with long-lasting effects for up to 28 days post-MI, indicating perturbation of CSF2 signalling as an important therapeutic intervention to dampen the inflammatory response and adverse cardiac remodelling post-MI.

**Figure 5:**
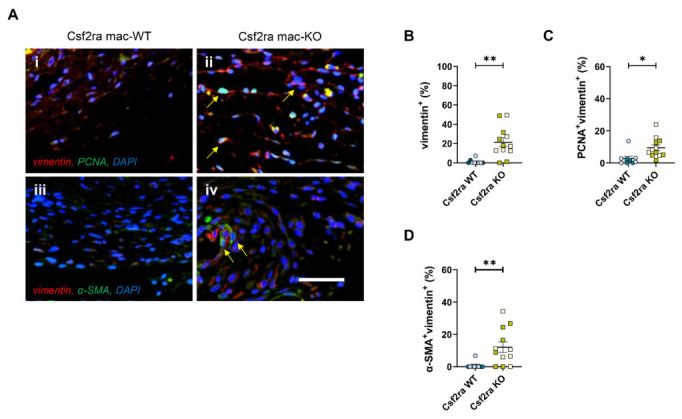
Macrophage-specific Csf2ra deficiency promotes CXCL10-positive macrophages without affecting CTSZ-positive macrophages post-myocardial infarction. Representative images of (**A**(**i**,**ii**)) PCNA^+^vimentin^+^ and (**A**(**iii**,**iv**)) α-SMA^+^vimentin^+^ cells in Csf2ra mac-WT (*n* = 9) and -KO mice (*n* = 12). Cells positive for both markers are observed as yellow/white with examples indicated by arrows, with DAPI used as a nuclear marker. Quantification of cardiac fibroblast (**B**) density, (**C**) proliferation, and (**D**) α-SMA protein expression. Statistical significance is reported as * *p* < 0.05 or ** *p* < 0.01, using an unpaired Student’s *t*-test. The scale bar represents 500 μm and is applicable to all panels. Csf2ra mac-WT male mice are presented with pale blue circles and female mice with blue circles; Csf2ra mac-KO male mice are presented with pale mustard squares and female mice with mustard squares.

Additionally, Csf2ra mac-KO infarcts displayed significantly increased cardiac fibroblast density (assessed by quantification of vimentin-positive cells, Figure 5A(i,ii),B), which was associated with augmented proliferation (Figure 5C) and a greater number of alpha-smooth muscle actin (α-SMA)-expressing cardiac fibroblasts (Figure 5A(iii,iv),D). Lastly, loss of Csf2ra in macrophages did not affect the number of capillaries or associated endothelial cells undergoing apoptosis 28 days post-MI. Collectively, our results indicate that macrophage-specific deficiency of Csf2ra enhances cardiac repair and function in response to injury through scar collagen maturation, in part due to abrogation of infiltrating macrophages acquiring a pro-inflammatory phenotype. Together these findings strengthen the rationale for our previously described specific Csf2ra inhibition as a therapeutic strategy to regulate the inflammatory response and promote cardiac repair in response to acute myocardial injury.

We have previously described sex-biassed effects following pharmacological inhibition of the CSF2RA inhibitor in mice post-MI, where we have shown that changes in echocardiographic and histological characteristics were more prominent in female mice [22]. Subsequently, we analysed sex-specific data in the macrophage-specific Csf2ra deficiency study (Table 4). In line with previous findings, macrophage-specific Csf2ra deficiency resulted in improved cardiac function and collagen maturation in female mice. Macrophage accrual was significantly increased in both male and female mice.

## 4. Discussion

Suppression of CSF2 signalling has emerged as a therapeutic intervention with direct translational potential to calibrate the post-MI inflammatory response and limit pathological cardiac remodelling [18,22]. In the present study, we identify macrophage-intrinsic CSF2RA signalling as a principal mediator of CSF2-driven effects and a key regulator of cardiac repair in post-MI remodelling. Using a tamoxifen-inducible macrophage-specific Csf2ra deletion model, we demonstrate that loss of Csf2ra in macrophages is associated with an early improvement in left ventricular function, in part by promoting the accumulation of macrophages with reparative-associated marker enrichment and accelerating collagen scar maturation. Importantly, the present study provides mechanistic support for, rather than direct therapeutic demonstration of, CSF2RA inhibition as a strategy to limit adverse remodelling post-MI.

Macrophages play a central role in coordinating wound healing and cardiac remodelling post-MI and their phenotype is tightly regulated by the local cytokine milieu [24]. Although CSF2 has previously been implicated in sustaining inflammatory macrophage activation, prior studies relied on global Csf2 or Csf2rb deficiency [18], limiting the ability to attribute the observed effects to macrophage-intrinsic signalling. By selectively ablating Csf2ra in macrophages, our data isolate the direct contribution of CSF2RA signalling to macrophage behaviour in the injured myocardium. The genetic macrophage-specific Csf2ra deletion shares several key phenotypic features with our prior pharmacological study employing a selective CSF2RA peptide inhibitor, E21R [22]. Both approaches produced an early improvement in left ventricular function, accumulation of macrophages with anti-inflammatory and reparative-associated marker enrichment, and enhanced collagen scar maturation. Additionally, we observed consistent sex-biassed effects that were more pronounced in female mice in both studies. Csf2ra mac-KO mice exhibited a significant increase in FS%, compared to WT controls, indicating improved contractility and cardiac function in the absence of macrophage CSF2 signalling. This effect was more pronounced in female Csf2ra mac-KO mice, consistent with our previous observations using pharmacological CSF2RA inhibition [22]. These concordant findings strengthen confidence that macrophage-intrinsic CSF2RA signalling contributes meaningfully to the post-MI reparative environment. However, the two approaches are not phenotypically identical, which is not uncommon in complex in vivo pathologies when utilising gene ablation methods in specific cell populations compared to systemic pharmacological inhibitory approaches. In the pharmacological study [22], CSF2RA inhibition limited the border-zone fibrosis and was mechanistically linked to reduced CTSZ expression and enhanced CXCL10 signalling. In the present genetic model, the phenotype is more restricted: while CXCL10-positive macrophage enrichment is reproduced, CTSZ abundance is not significantly altered, and there is no change in overall replacement or interstitial fibrosis burden. Regardless, macrophage-specific Csf2ra deficiency replicated the effect on collagen maturation: polarised light analysis of Picrosirius red-stained sections revealed an increased proportion of mature collagen fibres within the scar, indicative of enhanced fibrillar collagen network, which is associated with increased tensile strength of the ventricular myocardium and preserved structure [25,26]. The differences observed between the two studies could be attributed to the distinct nature of systemic CSF2RA inhibition, which could also affect other CSF2RA-expressing cells such as neutrophils, compared to the present genetic study that isolates the macrophage-intrinsic effects. Additionally, while our refined LAD ligation model improves animal welfare and reduces the number of animals used in research, the caveat is that it produces a restricted injury, which may be associated with less pernicious progression and thereby masks aspects of late-stage effects. Accordingly, the present genetic study replicates the CXCL10-related component of the previously proposed pathway and suggests that this effect relates to the macrophage-intrinsic CSF2RA signalling, without fully reproducing all previously reported molecular changes.

At a cellular level, macrophage-specific Csf2ra deficiency resulted in the accumulation of macrophages enriched in anti-inflammatory and pro-reparative markers, as evidenced by co-localisation with CD206 and CXCL10. The inflammatory chemokine CXCL10 was previously identified as a macrophage-secreted protein that is markedly reduced following CSF2 polarisation [22], thereby further strengthening the in vivo efficacy of macrophage-specific Csf2ra deletion. We have previously described a CTSZ/CXCL10-mediated mechanism identified through specific CSF2RA inhibition [22]. The present genetic study supports the CXCL10 component of the previously proposed mechanism; however, a significant reduction in CTSZ abundance was not replicated in the Csf2ra mac-KO mice. Additionally, CXCL10 is involved in the infiltration of CXCR3-positive fibroblasts in the injured myocardium, and CXCL10^−/−^ is associated with impaired recruitment of CXCR3-positive α-SMA-expressing fibroblasts [27]. As a result of the macrophage CXCL10 expression, the enhanced scar maturation observed in Csf2ra mac-KO mice was accompanied by increased fibroblast density, proliferation, and α-SMA expression, suggesting altered macrophage–fibroblast crosstalk. Pro-reparative macrophages are known to influence fibroblast activation and ECM remodelling through paracrine signalling [10], and our data support a model in which suppression of CSF2RA signalling promotes a macrophage phenotype that favours reparative wound healing.

Our study has several limitations. Firstly, while Csf2ra deficiency improved cardiac function and structure at 14 days post-MI, the effects were not persistent at 28 days post-MI, indicating that CSF2RA loss accelerates early reparative remodelling without conferring long-term protection. This disparity could be explained partly due to the use of a refined distal LAD ligation model which produces smaller infarcts and may have attenuated the magnitude of later divergence between groups. Importantly, the persistent changes in fibrillar collagen content, cellular composition, and macrophage populations observed at 28 days provide biological coherence despite the transient functional benefit, suggesting stage-specific effects on the reparative process. Secondly, we did not assess the indirect effects of Csf2ra deficiency upon cardiomyocyte survival and hypertrophy. Thirdly, validation of Csf2ra deletion was performed in peripheral blood mononuclear cells and adherent monocytes. Direct confirmation of deletion efficiency in infarct macrophages within the injured heart was not undertaken, and macrophage specificity in the cardiac context is therefore inferred from the Csf1r-Cre/Esr1 strategy and peripheral validation data.

Our findings are consistent with and extend previous reports, including ours, demonstrating improved cardiac remodelling following disruption of the CSF2/CSF2R axis [18,22]. Importantly, the concordance between genetic macrophage-specific Csf2ra deletion and prior pharmacological CSF2RA inhibition strengthens the translational relevance of this pathway. Together, these data support CSF2RA as a critical checkpoint for modulating macrophage phenotype during the post-MI inflammatory response and limiting adverse cardiac remodelling.

## 5. Conclusions

In summary, this study demonstrates that macrophage-intrinsic CSF2RA signalling is a key regulator of post-MI repair. Selective deletion of Csf2ra in macrophages improves cardiac function by promoting inflammatory resolution and enhancing collagen scar maturation without altering infarct size. These findings provide mechanistic support for CSF2RA as a promising therapeutic target to limit pathological cardiac remodelling and improve post-MI recovery.

## Figures and Tables

**Table 1:** Primary antibodies used for immunohistochemistry.

Antibody	Working Conc.	Cat. No.; Supplier
anti-α-SMA	2.66 μg/mL	A2547; Sigma-Aldrich
anti-CD68	0.33 μg/mL	97778; CST
anti-CD68	1.56 μg/mL	M0814; Dako
anti-CD206	0.31 μg/mL	24595; CST
anti-CTSZ	2.0 μg/mL	AF1033; R&D Systems
anti-CXCL10	6.67 μg/mL	Ab9807; Abcam
anti-iNOS	0.9 μg/mL	68186; CST
anti-PCNA	3.27 μg/mL	M0879; Dako
anti-vimentin	2.5 μg/mL	Ab92547; Abcam

**Table 2:** Echocardiographic assessment of cardiac function of Csf2ra mac-WT (*n* = 13) and -KO mice (*n* = 11) at 14 days post-myocardial infarction. Statistical significance is reported as * *p* < 0.05, ** *p* < 0.01, or *** *p* < 0.001, using an unpaired Student’s *t*-test.

Day 14 Post-MI	WT	KO	*p*-Value
IVS; d (mm)	0.93 ± 0.05	0.95 ± 0.06	0.8278
IVS; s (mm)	1.24 ± 0.04	1.26 ± 0.08	0.7777
LVID; d (mm)	3.84 ± 0.08	3.77 ± 0.08	0.5663
LVID; s (mm)	2.87 ± 0.10	2.63 ± 0.12	0.1341
LVPW; d (mm)	0.83 ± 0.04	1.20 ± 0.09	0.0009 ***
LVPW; s (mm)	1.17 ± 0.05	1.53 ± 0.08	0.0011 **
EF (%)	50.45 ± 2.44	58.42 ± 2.77	0.0497 *
FS (%)	25.43 ± 1.45	30.63 ± 1.92	0.0470 *
LV Vol; d (μL)	63.92 ± 2.97	61.25 ± 3.18	0.5632
LV Vol; s (μL)	32.13 ± 2.51	26.17 ± 2.95	0.1518

IVS, interventricular septum; LVID, left ventricular internal diameter; LVPW, left ventricular posterior wall thickness; EF, ejection fraction; FS, fractional shortening; LV Vol, left ventricular volume; d, diastole; s, systole.

**Table 3:** Echocardiographic assessment of cardiac function of Csf2ra mac-WT (*n* = 10) and -KO mice (*n* = 10) at 28 days post-myocardial infarction. Statistical significance was tested using an unpaired Student’s *t*-test.

Day 28 Post-MI	WT	KO	*p*-Value
IVS; d (mm)	0.98 ± 0.05	1.05 ± 0.09	0.5661
IVS; s (mm)	1.24 ± 0.07	1.32 ± 0.10	0.4775
LVID; d (mm)	4.22 ± 0.35	4.05 ± 1.25	0.3678
LVID; s (mm)	3.20 ± 0.31	2.96 ± 0.16	0.3182
LVPW; d (mm)	1.02 ± 0.13	1.03 ± 0.09	0.9094
LVPW; s (mm)	1.24 ± 0.16	1.38 ± 0.07	0.3200
EF (%)	48.15 ± 5.38	53.08 ± 3.19	0.3197
FS (%)	24.29 ± 2.86	27.32 ± 1.98	0.3103
LV Vol; d (μL)	80.38 ± 8.95	73.11 ± 5.27	0.3752
LV Vol; s (μL)	42.50 ± 6.21	35.80 ± 4.79	0.3740

IVS, interventricular septum; LVID, left ventricular internal diameter; LVPW, left ventricular posterior wall thickness; EF, ejection fraction; FS, fractional shortening; LV Vol, left ventricular volume; d, diastole; s, systole.

**Table 4:** Echocardiographic and histological assessment of Csf2ra mac-WT and -KO mice segregated by sex. Statistical significance is reported as * *p* < 0.05, using an unpaired Student’s *t*-test.

	Male	Female
FS (%) (day 14)	0.5515	0.0346 *
Replacement fibrosis (μm^2^)	0.3163	0.8956
Interstitial fibrosis (%)	0.5181	0.4667
Fibrillar collagen fibre content (%)	0.2168	0.0130 *
Granularity (cell/mm^2^)	0.4474	0.6016
CD68^+^ (%)	0.0218 *	0.0195 *
PCNA^+^CD68^+^ (%)	0.5170	0.7308
CD206^+^CD68^+^ (%)	0.0267 *	0.1520
iNOS^+^CD68^+^ (%)	0.6116	0.0415 *
CXCL10^+^CD68^+^ (%)	0.3893	0.0669
CTSZ^+^CD68^+^ (%)	0.1588	0.9400
Vimentin^+^ (%)	0.0129 *	0.0705
PCNA^+^vimentin^+^ (%)	0.0937	0.0327 *
α-SMA^+^vimentin^+^ (%)	0.1005	0.0662

## Data Availability

The original contributions presented in this study are included in the article. Further inquiries can be directed to the corresponding author.

## Abbreviations

The following abbreviations are used in this manuscript:

α-SMA	Alpha-smooth muscle actin
CSF	Colony-stimulating factor
Csf2ra mac-KO	Csf2ra macrophage-specific deficiency
Csf2ra mac-WT	Csf2ra macrophage wild type
CSF2RA/B	CSF2 receptor subunit alpha/beta
CTSZ	Cathepsin Z
CXCL10	CXC Motif Chemokine Ligand 10
ECM	Extracellular matrix
EF	Ejection fraction
FS	Fractional shortening
GM-CSF	Granulocyte macrophage colony-stimulating factor
LAD	Left anterior descending
LVPW	Left ventricular posterior wall
MI	Myocardial infarction
